# Combination Therapy with Trehalose and Hyaluronic Acid Restores Tear Lipid Layer Functionality by Ameliorating Inflammatory Response Protein Markers on the Ocular Surface of Dry Eye Patients

**DOI:** 10.3390/jcm14155525

**Published:** 2025-08-05

**Authors:** Natarajan Perumal, Caroline Manicam, Eunjin Jeong, Sarah Runde, Norbert Pfeiffer, Franz H. Grus

**Affiliations:** Experimental and Translational Ophthalmology, Department of Ophthalmology, University Medical Centre of the Johannes Gutenberg University Mainz, Langenbeckstr. 1, DE-55131 Mainz, Germany

**Keywords:** dry eye disease, proteomics, inflammation, tears, trehalose, hyaluronic acid

## Abstract

**Objectives:** Topical lubricants are the fundamental treatment for dry eye disease (DED). However, the molecular mechanisms underlying their efficacy remain unknown. Here, the protective effects of Thealoz^®^ Duo with 3% trehalose and 0.15% hyaluronic acid are investigated in DED patients by a longitudinal clinical study and subsequent elucidation of the tear proteome and cell signaling changes. **Methods:** Participants were classified as moderate to severe DED (DRY, *n* = 35) and healthy (CTRL, *n* = 23) groups. Specific DED subgroups comprising evaporative (DRYlip) and aqueous-deficient with DRYlip (DRYaqlip) were also classified. Only DED patients received Thealoz^®^ Duo. All participants were clinically examined before (day 0, T1) and after the application of Thealoz^®^ Duo at day 28 (T2) and day 56 (T3). Next, 174 individual tear samples from all groups at three time-points were subjected to proteomics analysis. **Results:** Clinically, Thealoz^®^ Duo significantly improved the ocular surface disease index at T2 vs. T1 (DRY, *p* = 1.4 × 10^−2^; DRYlip, *p* = 9.2 × 10^−3^) and T3 vs. T1 (DRY, *p* = 2.1 × 10^−5^; DRYlip, *p* = 1.2 × 10^−4^), and the tear break-up time at T3 vs. T1 (DRY, *p* = 3.8 × 10^−2^; DRYlip, *p* = 1.4 × 10^−2^). Thealoz^®^ Duo significantly ameliorated expression of inflammatory response proteins (*p* < 0.05) at T3, which was observed at T1 (DRY, *p* = 3.4 × 10^−4^; DRYlip, *p* = 7.1 × 10^−3^; DRYaqlip, *p* = 2.7 × 10^−8^). Protein S100-A8 (S100A8), Alpha-1-antitrypsin (SERPINA1), Annexin A1 (ANXA1), and Apolipoprotein A-I (APOA1) were found to be significantly reduced in all the DED subgroups. The application of Thealoz^®^ Duo showed the therapeutic characteristic of the anti-inflammatory mechanism by promoting the expression of (Metalloproteinase inhibitor 1) TIMP1 in all the DED subgroups. **Conclusions:** Thealoz^®^ Duo substantially improved the DED symptoms and restored the functionality of the tear lipid layer to near normal in DRYlip and DRY patients by ameliorating inflammation. Notably, this study unravels the novel mechanistic alterations underpinning the healing effects of Thealoz^®^ Duo in DED subgroups in a time-dependent manner, which supports the improvement in corresponding clinical attributes.

## 1. Introduction

Dry eye disease (DED) is a term used to collectively define a pathology of the ocular surface with pleiotropic etiology that affects the tear film homeostasis, causing deleterious inflammatory changes and discomfort of varying degrees and pain, which ultimately results in perturbations in the vision function [1]. While women and individuals over 50 years of age are highly predisposed towards the development of this common yet debilitating ocular disease, there has been a notable increase in incidences in younger individuals of both genders recently, presumably due to the growing use of digital devices, making DED a widespread and growing healthcare concern [2].

The increasing number of DED incidences has expanded the global market for topical eye drops, which are readily available as over-the-counter (OTC) medications [3]. Topical administration of eye drops consisting of a variety of demulcents, with and without preservatives, that can be used directly on the ocular surface remains the fundamental drug delivery system for the treatment of DED [4,5]. However, the current challenge is the successful treatment of the various manifestations of the disease that can be divided into specific DED subgroups as a consequence of aqueous tear-deficiency (DRYaq) or due to changes in the lipid phase (DRYlip) of the tear film. In the line of commercially available eye drops for DED, Thealoz^®^ Duo eye drops (Laboratoires Thea, Clermont-Ferrand, France) containing 3% trehalose and 0.15% sodium hyaluronate has emerged as a promising product in the market due to its profound effect on ocular surface homeostasis recovery [6]. Trehalose is a naturally occurring non-reducing disaccharide of glucose molecules with exceptional stability and thus is an important energy source in most organisms with myriad bioprotective features, namely protection from desiccation, oxidative damage, and other exogenous stressors [7,8,9,10]. Owing to its unique inherent properties, this simple sugar has been widely used in the formulation of commercialized pharmaceutical products [11]. Taking advantage of this multifunctional natural product, many studies have evaluated its clinical efficacy in recent years, such as the clinical study by Doan et al., which proved that the use of an eye drop solution containing trehalose in addition to hyaluronic acid improved the subjective ocular symptoms of DED patients after 84 days of use [12]. Similarly, the use of Thealoz^®^ Duo eye drops was demonstrated to significantly improve DED symptoms in peri- and post-menopausal women after three months [13]. Additionally, the positive outcome of the use of Thealoz^®^ Duo was also reflected in increased tear film thickness and retention time on the ocular surface [14]. Fariselli et al. documented improvement in the tear break-up time (TBUT) of the lipid layer, as well as DED symptom attributes, along with increased goblet cell density in the evaporative DED subgroup after the use of Thealoz^®^ Duo for a duration of two months [15]. Interestingly, recent microscopy-based evidence on the cellular effects of hyaluronic acid (HA) and trehalose promotes their role in conjunctival microvilli regeneration and epithelial protection. Notably, the prolonged administration made it possible to obtain a notable increase in the concentration of microvilli and an improvement or complete normalization of the microscopic picture of the ocular surface [16]. These findings provide important mechanistic support for the use of these compounds in dry eye therapy. Although these exciting studies have all provided compelling evidence on the efficacy and significant improvement in the clinical parameters attributed to the DED, the underlying intricate molecular mechanisms on the ocular surface and in tears during or after the instillation of Thealoz^®^ Duo remain largely unknown.

Therefore, this study took advantage of the state-of-the-art mass spectrometry (MS)-based proteomics strategy in combination with clinical diagnosis to elucidate the protective mechanisms underpinning the use of Thealoz^®^ Duo eye drops. The hallmark of this study is that different DED subgroups were distinctly categorized and investigated by longitudinal clinical examinations prior to subjecting individual tear samples of DED patients to in-depth proteomics and bioinformatics analyses.

## 2. Materials and Methods

### 2.1. Subjects

This clinical study is an open, non-randomized, longitudinal, and monocentric study. The clinical study protocols and design were approved by the local ethics committee of the Medical Board of Rhineland-Palatinate, Germany (Landesärztekammer Rheinland-Pfalz; application number 2018-13625-clinical research). This clinical study was conducted in accordance with the Declaration of Helsinki adopted by the World Medical Assembly in June 1964, and last amended in Fortaleza, Brazil, 2013, and according to the ICH E6 guidelines for good clinical practice. In accordance with the Declaration of Helsinki, all subjects were informed about the risks, privacy policy, and the general aim of this study. Written informed consents were obtained from all study participants before initiating any clinical procedures. Subjects were recruited by the Department of Ophthalmology at the University Medical Center of the Johannes Gutenberg-University, Mainz.

Briefly, all study participants underwent thorough diagnostic steps according to distinct inclusion and exclusion criteria. These consisted of basic secretory tests (BST), tear break-up time (TBUT), and refraction assessments, as well as symptom anamneses employing the ocular surface disease index (OSDI) and visual analog scale (VAS; symptoms of eye complaints) questionnaires. The VAS was acquired from the participants based on a linear indication of ocular discomfort between 0 and 100. The symptoms questioned were as follows: foreign body sensation, dryness of the eyes, sticky feeling, itching, photophobia (sensitive to light), pain, blurred vision, and burning/tingling sensation. The visus and visual refraction based on the sphere (near-sighted/far-sighted), cylinder (astigmatism), and axis were also evaluated.

A total of 58 participants were classified and divided into moderate to severe DED (DRY, *n* = 35) and healthy (CTRL, *n* = 23) groups. Further DRY subgroups consisting of patients with evaporative DED (DRYlip, *n* = 15) and severe aqueous-deficient with evaporative DED (DRYaqlip, *n* = 12) were also clinically identified and classified accordingly. The designated groups were classified based on the following clinical attributes: DRY = TBUT ≤ 10 s or BST with Schirmer I test ≤ 5 mm and ≥2 mm or OSDI ≥ 22; DRYlip = TBUT ≤ 10 s and BST with Schirmer I test > 10 mm and OSDI ≥ 22; DRYaqlip = TBUT ≤ 10 s and BST with Schirmer I test ≤ 5 mm and ≥ 2 mm and OSDI ≥ 22 (Supplementary Data S1). Individuals without any of the aforementioned ophthalmic attributes were classified as healthy CTRL group. The eye with the worst symptoms was chosen for subsequent clinical and proteomics analysis following a pre-screening clinical assessment of both eyes of all patients. Participants were excluded if one or more of the following aspects applied to them: any ocular pathology judged by the investigator as incompatible with the study and/or any other clinically relevant ocular abnormality except dry eye disease, history of allergy, known hypersensitivity to one or more component(s) of the medical product, pregnancy, and lactation. Similarly, excluded individuals were also those who had participated in another clinical study within the 4 weeks before and during the study period.

### 2.2. Study Design

A total of 3 visits were scheduled for all the participants in both groups. Visit 1 (T1) is the baseline study day (day 0). Participants were not allowed to use any topical lubricants for 12–24 h before the study day. Baseline measurements of all clinical parameters were taken. Only participants in the DED group were given Thealoz^®^ Duo (Laboratoires Thea, France) eye drops for administration (1 drop in each eye, 4 to 6 times a day). They were also provided with a diary in which they documented the frequency and time-points of product instillation. A total treatment period of 56 days was scheduled. All clinical parameters were measured all over again at visit 2 (day 28 ± 4; T2) and 3 (day 56 ± 4; T3) for participants in both groups.

### 2.3. Clinical Data Analysis

Descriptive statistical analysis to assess differences in all the clinical attributes was conducted with Statistica version 13 software for analysis of variance (ANOVA) and the post-hoc Tukey honest significant difference (HSD) test for unequal N (Spjotvoll/Stoline test). Clinical attributes were found to be significant with a *p*-value < 0.05 (Supplementary Data S2). Box plots of the clinical attributes of the designated groups were generated.

### 2.4. Tear Sample Collection and Preparation for Proteomics Analysis

Tear samples were collected from all subjects at the different time-points using the Schirmer technique, as described in our previous studies, sans the use of topical anesthetic eye drops [17,18]. Collected Schirmer strips (Schirmer Tear Test, Optitech Eyecare, Uttar Pradesh, India) were placed in 2 mL safe-lock Eppendorf microtubes and immediately frozen at −80 °C until use. In order to extract tear proteins from the Schirmer strips, 200 µL (BST ≤ 5 mm) or 300 µL (BST > 5 mm) phosphate-buffered saline (PBS) was added to the strips and shaken for 3 h by an IntelliMixer at 4 °C [17]. Subsequently, protein concentration was measured with the bicinchoninic acid (BCA) protein assay kit (Pierce, Rockford, IL, USA) prior to in-solution trypsin digestion and peptide purification with SOLAµ™ SPE HRP plates (Thermo Fisher Scientific, Rockford, IL, USA) according to the manufacturer’s instructions. The resulting peptide eluate was concentrated to dryness in a centrifugal vacuum evaporator and dissolved in 0.1% formic acid with the final protein concentration of 250 ng/µL.

### 2.5. Mass Spectrometry (MS)-Based Clinical Proteomics Analysis

The nano-liquid chromatography (nLC)-MS system employed comprised an EASY-nLC 1200 system (Thermo Scientific, Rockford, IL, USA) with an Acclaim PepMap RSLC, 75 µm × 50 cm, nanoViper analytical column (Thermo Scientific, Rockford, IL, USA) directly coupled to ESI-LTQ-Orbitrap-XL MS (Thermo Scientific, Bremen, Germany), as described elsewhere [19,20,21,22]. A total of 2 µL of each sample (500 ng) was used to fractionate peptides at a flow of 300 µL/min. Solvent A was LC-MS grade water with 0.1% (*v*/*v*) formic acid, and solvent B consisted of LC-MS grade acetonitrile with 20% (*v*/*v*) water and 0.1% (*v*/*v*) formic acid. The run of the resulting gradient per sample added up to a total time of 120 min; 0–90 min: 5–30% B, 90–100 min: 30–100% B, 100–120 min: 100% B. Briefly, the LTQ-Orbitrap was operated in a data-dependent mode of acquisition and survey full scan MS spectra from *m*/*z* 300 to 2000 were acquired in the Orbitrap with a resolution of 60,000 at *m*/*z* 400 and a target automatic gain control setting of 1.0 × 10^6^ ions. The five most intense precursor ions were sequentially isolated for fragmentation and recorded in the LTQ.

### 2.6. Label-Free Quantitative Proteomics Analysis

The acquired continuum MS spectra were analyzed utilizing MaxQuant version 1.6.17.0 software [23,24,25,26,27]. The tandem MS spectra were searched against the *Homo sapiens* database [Uniprot, reviewed (Swiss-Prot); Accession date, 16 August 2020; Annotated proteins, 20,385] with a target/decoy-based FDR for peptide and protein identification set to 0.01. The peptide sequences of the three isoforms of proline-rich protein 4 (PRR4), which is a key protein implicated in the DED, were used as described in our previous study [28]. The summary of MaxQuant parameters employed in the current analysis is tabulated in Supplementary Data S3a. The generated protein list based on the iBAQ (intensity-Based Absolute Quantification) algorithm from the MaxQuant analysis was used for subsequent statistical analysis with Perseus version 1.6.14.0 software [29]. First, a log_2_ transformation of all protein intensities was conducted, and results were filtered to only include peptides with 70% valid measured values in at least one of the study groups. Missing values were subsequently imputed from a normal distribution in standard settings (width: 0.2, down shift: 1.8), enabling statistical analysis [27]. For statistical evaluation, a Student’s two-sided *t*-test was used for all the groups’ comparison with *p* ˂ 0.05 to identify the significantly differentially abundant proteins. Unsupervised hierarchical clustering analysis of the identified differentially abundant proteins was performed according to Euclidean distance (Linkage, Average; Constraint, preserve order; Preprocess with k-means, enabled; Number of clusters, 300; Maximal number of iterations, 10; Number of restarts, 1). Box plots of the selected differentially abundant proteins (DAP) were generated via Statistica version 13.

### 2.7. Functional Annotation and Pathway Analyses

The list of the significantly differentially abundant proteins in the designated comparison groups was used for functional annotation analysis employing Ingenuity Pathway Analysis (IPA) [30]. The top enriched terms of the gene ontology cellular component (GOCC), molecular types, biological functions, and canonical pathways of the differentially abundant proteins were presented with *p*-values calculated using Benjamini–Hochberg (B-H) multiple testing correction (one-sided Fisher’s exact test, −log B-H *p*-value > 1.3). Unsupervised hierarchical clustering analysis of the top canonical pathways and biological functions, as well as the annotated differentially abundant proteins, were performed according to Euclidean distance (Linkage, average; Constraint, preserve order; Preprocess with k-means, enabled; Number of clusters, 300; Maximal number of iterations, 10; Number of restarts, 1) utilizing Perseus software version 1.6.14.0.

## 3. Results

### 3.1. Clinical Investigation on the Effects of Thealoz^®^ Duo Eye Drops on the Ocular Surface

**Figure 1:**
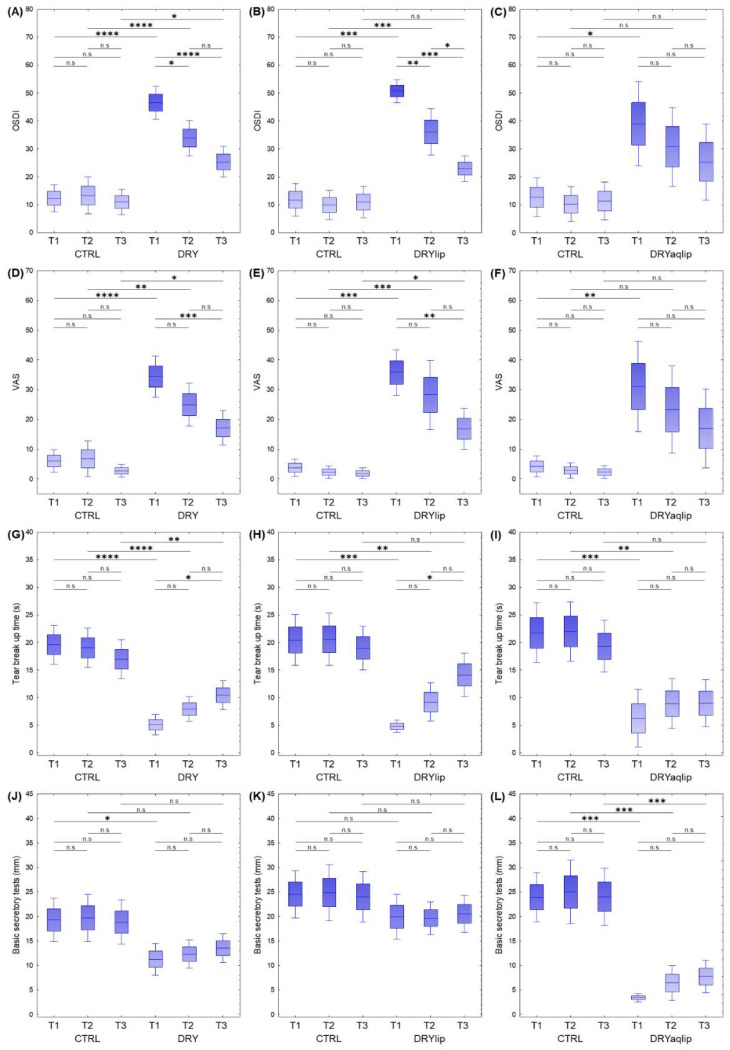
Box plots show the results of the significant changes in the clinical attributes (*p* < 0.05) comprising the (**A**–**C**) Ocular Surface Disease Index (OSDI) Questionnaire score, (**D**–**F**) Visual Analog Scale (VAS), (**G**–**I**) Tear Breakup Time (TBUT), and (**J**–**L**) Basic Secretory Test (BST) in different dry eye (DED) subgroups compared to healthy controls (CTRL). The *y*-axis represents the values in each individual. Boxes represent the mean ± SE and mean ± 1.96SE. * *p* < 0.05; ** *p* < 0.001; *** *p* < 0.0001; **** *p* < 0.00001; n.s: non-significant.

Tear samples from a total of 35 DRY (age: 57.60 ± 2.48; gender: 22 female and 13 male) and 23 CTRL (age: 46.52 ± 3.06; gender: 13 female and 10 male) subjects were used in this study (Table 1, Supplementary Data S1). Further categorization of the DRY patients based on distinct clinical attributes resulted in two different DED subgroups composed of DRYlip (*n* = 15) and DRYaqlip (*n* = 12), which were also subjected to the evaluation of the efficacy of Thealoz^®^ Duo at different time-points. The complete results of the statistical analysis of the clinical attributes of the designated DED subgroups compared to CTRL are tabulated in Supplementary Data S2. The outcomes of the comprehensive clinical statistical analyses of the BST, TBUT, OSDI, and VAS scores and other clinical parameters such as the visus and the refraction (sphere, axis and cylinder) attributes demonstrated no significant differences in all CTRL groups at all different time-points, which supports the robustness of our patient classification strategy (Figure 1, Supplementary Data S2). The OSDI score was found to be significantly increased in all DED subgroups compared to their respective CTRL at the T1 (DRY vs. CTRL, *p* = 2.0 × 10^−5^; DRYlip vs. CTRL, *p* = 1.2 × 10^−4^; DRYaqlip vs. CTRL, *p* = 2.11 × 10^−2^) (Figure 1A–C). Remarkably, a general pattern of gradual decrement in the mean OSDI score was observed in all DRY subgroups following the acute and chronic treatment with Thealoz^®^ Duo eye drops (Figure 1A–C)**.** In the first group represented by the DRY group, the significant improvement in the OSDI score is reflected in DRY vs. CTRL at T2 (*p* = 4.7 × 10^−5^) and at T3 (*p* = 1.7 × 10^−2^) (Figure 1A). In short, the degree of improvement in the OSDI score was higher at T3 (DRY_T3 vs. DRY_T1, *p* = 2.1 × 10^−5^) compared to T2 (DRY_T2 vs. DRY_T1, *p* = 1.4 × 10^−2^).

Similar significant improvement in the OSDI attribute was also observed in the DRYlip vs. CTRL at T2 (*p* = 1.2 × 10^−4^) and remarkably, the OSDI score was near normal at T3 (*p* > 0.05), which corresponded to a higher degree of improvement in the OSDI at T3 (DRYlip_T3 vs. DRYlip_T1, *p* = 1.2 × 10^−4^) than at T2 (DRYlip_T3 vs. DRYlip_T1, *p* = 9.2 × 10^−3^) (Figure 1B). These results were the first line of clinical evidence that supports the efficacy of Thealoz^®^ Duo eye drops, which is significantly correlated with increased frequency of application to improve dry eye-related symptoms. On the contrary, no significant OSDI score differences were observed in the DRYaqlip subgroup at all three time-points apart from a slight improvement in the mean score value from 39.0 ± 7.7 at T1 to 25.4 ± 6.9 at T3 after the application of Thealoz^®^ Duo (Figure 1C). A similar pattern of time- and treatment-dependent decrement in the VAS measurements was also found in the DED subgroups (Figure 1D–F).

At the T1 time-point, the VAS measurements were recorded to be highly significant in all DED subgroups compared to their respective CTRL counterparts (DRY vs. CTRL, *p* = 2.0 × 10^−5^; DRYlip vs. CTRL1, *p* = 1.2 × 10^−4^; DRYaqlip vs. CTRL, *p* = 8.0 × 10^−3^). However, the mean VAS values were only significantly decreased at T3 compared to T1 in the DRY (DRY_T3 vs. DRY_T1, *p* = 4.1 × 10^−4^) and DRYlip (DRYlip_T3 vs. DRYlip_T1, *p* = 1.9 × 10^−3^) subgroups (Figure 1D,E). On the other hand, no significant differences (*p* > 0.05) were observed in the DRYaqlip subgroup at the different time-points, albeit a negligible decreasing trend from T1 to T3 (Figure 1F).

Next, we investigated the changes in the TBUT and found this clinical attribute to be significantly decreased in all DED subgroups compared to their respective CTRL at T1 (DRY vs. CTRL, *p* = 2.0 × 10^−5^; DRYlip vs. CTRL, *p* = 1.3 × 10^−4^, and DRYaqlip vs. CTRL, *p* = 7.0 × 10^−4^) (Figure 1G–I). The mean TBUT value in the DRY was significantly increased only at T3 in both DRY (DRY_T3 vs. DRY_T1, *p* = 3.8 × 10^−2^; Figure 1G) and DRYlip subgroups (DRYlip _T3 vs. DRYlip _T1, *p* = 1.4 × 10^−2^; Figure 1H) after Thealoz^®^ Duo usage. However, no significant differences (*p* > 0.05) were observed in the DRYaqlip subgroup at all different time-points, which showed no improvement in this clinical parameter even after the administration of the eye drops (Figure 1I).

Finally, we evaluated the potential changes in the BST attribute by measuring the Schirmer wetting length in mm. Interestingly, in contrast to all the other clinical diagnostic tests results in this study, the BST was overall not significantly affected, except for the significant changes in the DRY and DRYaqlip subgroups compared to their respective CTRL at baseline (DRY_T1 vs. CTRL_T1, *p* = 3.4 × 10^−2^; DRYlip_T1 vs. CTRL_T1, *p* > 0.05; DRYaqlip_T1 vs. CTRL_T1, *p* = 1.3 × 10^−4^) (Figure 1J–L). Generally, no significant differences in BST attributed to the use of Thealoz^®^ Duo were observed in all the DED subgroups at the different time-points.

### 3.2. Proteomics Investigation

**Figure 2:**
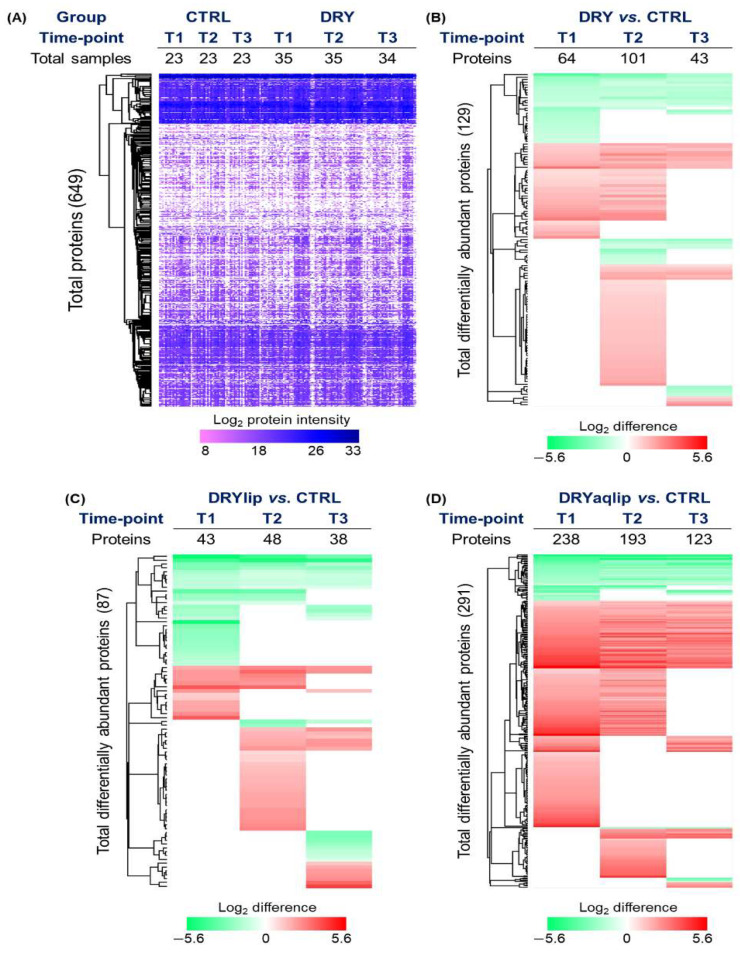
(**A**) Heat map depicts the hierarchical clustering of all 649 identified proteins from 173 tear samples based on the log_2_ protein intensity related to the designated groups. Different color intensities correspond to the degree of abundance. The hierarchical clustering of the significantly differentially abundant tear proteins identified based on the log_2_ difference in the designated dry eye (DED) subgroups compared to healthy controls (CTRL) at different time-points is depicted in the heat maps (**B**) moderate to severe DED (DRY) vs. CTRL, (**C**) evaporative DED (DRYlip) vs. CTRL, and (**D**) severe aqueous-deficient with evaporative DED (DRYaqlip) vs. CTRL. Up-regulated proteins are shown in red, and the down-regulated proteins are in green. Different color intensities correspond to the degree of log_2_ difference. Different time-points (T1, baseline), (day 28 ± 4; T2) and (day 56 ± 4; T3).

Label-free quantification analysis identified a total of 649 tear proteins (Figure 2A and Supplementary Data S3). In the DRY vs. CTRL, as many as 64, 101, and 43 proteins were significantly differentially abundant at T1, T2, and T3, respectively (Figure 2B and Supplementary Data S4a).

**Figure 3:**
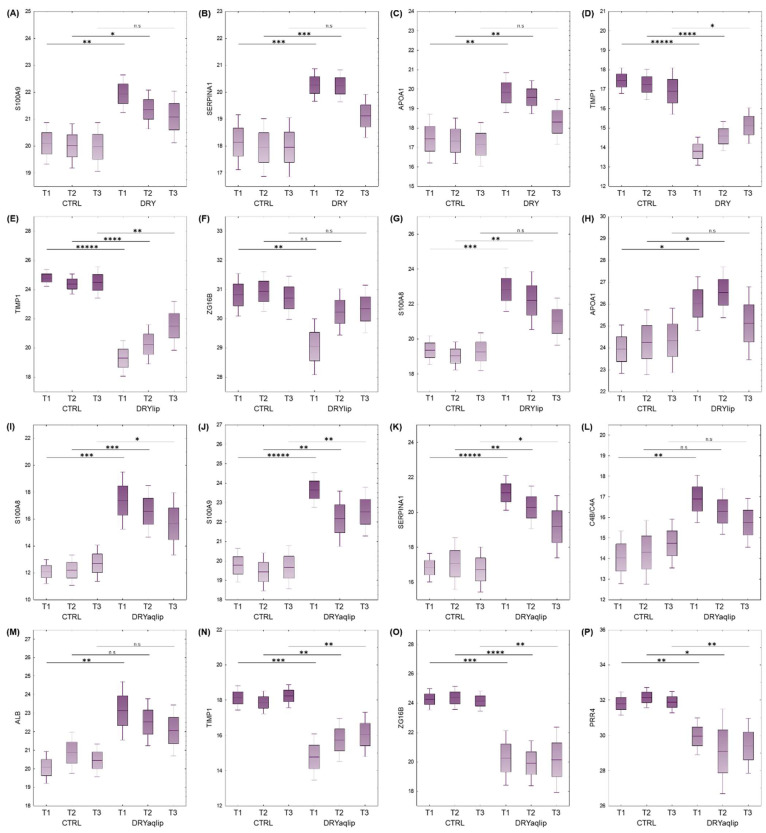
Box plots show the significantly differentially abundant protein profiles in the moderate to severe DED (DRY), evaporative DED (DRYlip) and severe aqueous-deficient with evaporative DED (DRYaqlip) compared to healthy controls (CTRL) at different time-points ((T1, baseline), day 28 ± 4; T2) and 3 (day 56 ± 4; T3)) following proteomics analysis: (**A**) Protein S100-A9 (S100A9), (**B**) Alpha-1-antitrypsin (SERPINA1), (**C**) Apolipoprotein A-I (APOA1), (**D**) Metalloproteinase inhibitor 1 (TIMP1), (**E**) TIMP1, (**F**) Zymogen granule protein 16 homolog B (ZG16B), (**G**) Protein S100-A8) (S100A8), (**H**) APOA1, (**I**) S100A8, (**J**) S100A9, (**K**) SERPINA1, (**L**) Complement C4-B/C4A (C4B/C4A), (**M**) Serum albumin (ALB), (**N**) TIMP1, (**O**) ZG16B, and (**P**) Proline-rich protein 4 (PRR4). The y-axis represents the intensities of the proteins in each individual sample. Boxes represent the mean ± SE and mean ± 1.96SE. * *p* < 0.05; ** *p* < 0.001; *** *p* < 0.0001; **** *p* < 0.00001; n.s: non-significant.

**Figure 4:**
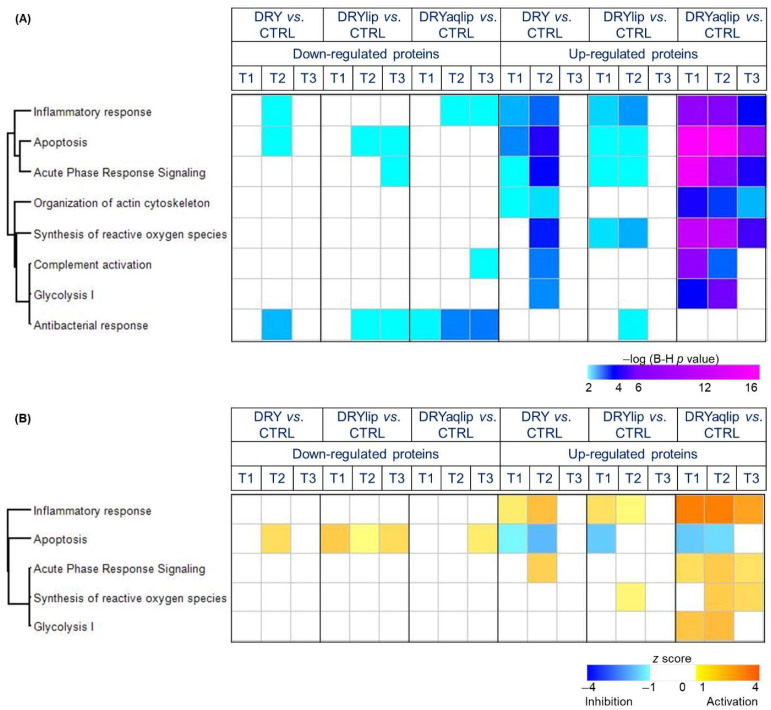
Hierarchical clustering of the significant biological functions and canonical pathways associated with the DAP in the designated DED groups at different time-points. (**A**) The significance of enrichment *p*-value was calculated using Benjamini–Hochberg (B-H) multiple testing correction (one-sided Fisher’s exact test, −log B-H *p*-value > 1.3) and is scaled by color intensity. (**B**) Overall z-scores are represented by the color orange, which indicates activation, and blue indicates inhibition of the signaling pathways/biological functions. Abbreviations: moderate to severe DED (DRY); subgroups: evaporative DED (DRYlip) and severe aqueous-deficient with evaporative DED (DRYaqlip), control groups (CTRL), different time-points (T1, baseline), (day 28 ± 4; T2) and (day 56 ± 4; T3).

It is noteworthy that a large number of proteins observed to be differentially abundant in the DRY vs. CTRL at T1 and T2 was found to be non-significant at T3, namely Protein S100-A9 (S100A9) (Figure 3A), Alpha-1-antitrypsin (SERPINA1) (Figure 3B), Apolipoprotein A-I (APOA1) (Figure 3C), and Metalloproteinase inhibitor 1 (TIMP1) (Figure 3D). In the DRYlip subgroup, there is generally a lower number of differentially abundant proteins (DAPs) at all time-points compared to the other two DED groups, with 43, 48, and 38 proteins identified at T1, T2, and T3, respectively (Figure 2C and Supplementary Data S4b). It is noteworthy that numerous DAPs observed in DRYlip vs. CTRL at T1 and T2 were found to be non-significant or significantly ameliorated at T3, namely TIMP1 (Figure 3E), Zymogen granule protein 16 homolog B (ZG16B) (Figure 3F), Protein S100-A8 (S100A8) (Figure 3G), and APOA1 (Figure 3H). The most profound effect of the Thealoz^®^ Duo eye drops was observed in the DRYaqlip vs. CTRL, which was reflected in the highest number of DAPs identified with 238, 193, and 123 proteins at T1, T2, and T3, respectively (Figure 2D and Supplementary Data S4c). Intriguingly, the expression of a cluster of 125 proteins, which were significantly up-regulated at T1 were significantly ameliorated at T3, namely S100A8 (Figure 3I), Protein S100-A9 (S100A9) (Figure 3J), Alpha-1-antitrypsin (SERPINA1) (Figure 3K), Complement C4-B/C4A (C4B/C4A) (Figure 3L), and Serum albumin (ALB) (Figure 3M). Notably, the lacrimal-specific proteins, which were decreased in abundance at T1, namely ZG16B (Figure 3O), Proline-rich protein 4 (PRR4) (Figure 3P), Lysozyme C (LYZ), Prolactin-inducible protein (PIP), Lipocalin-1 (LCN1), and Ig alpha-1 chain C region (IGHA1) were not influenced by the application of the Thealoz^®^ Duo eye drops at both time-points. In all dry eye subgroups, the inflammatory response was significantly activated at T1, with the highest degree of activation in the DRYaqlip subgroup (DRY: *z*-score = 1.5; DRYlip: *z*-score = 1.7; DRYaqlip: *z*-score = 4.0) (Figure 4 and Supplementary Data S5).

**Figure 5:**
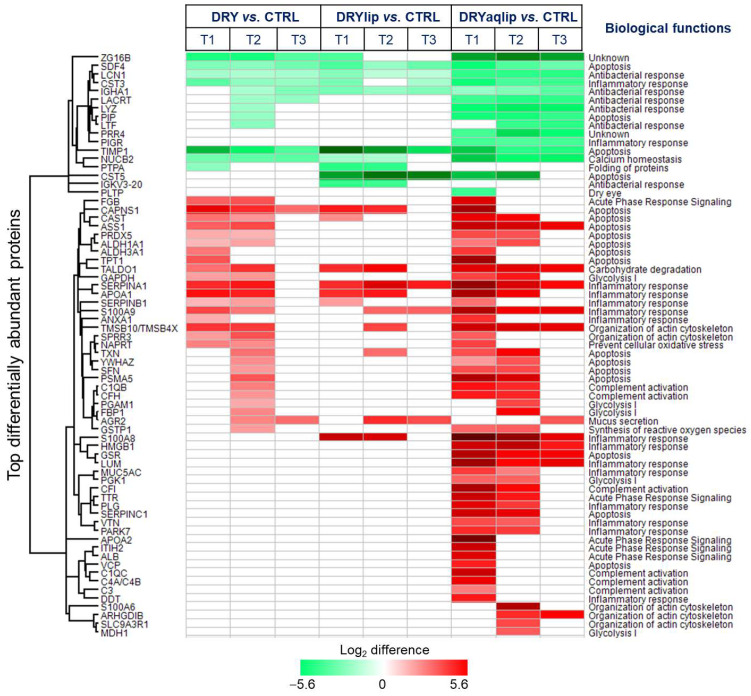
Heat map depicts the hierarchical clustering of the biological functions of the top significantly differentially abundant tear proteins identified based on the log_2_ difference in the designated dry eye (DED) subgroups compared to control (CTRL) at different time-points. Up-regulated proteins are shown in red, and the down-regulated proteins are in green. Different color intensities correspond to the degree of log_2_ difference. Abbreviations: moderate to severe DED (DRY); subgroups: evaporative DED (DRYlip) and severe aqueous-deficient with evaporative DED (DRYaqlip), control groups (CTRL), different time-points (T1, baseline), (day 28 ± 4; T2) and (day 56 ± 4; T3).

Remarkably, the continuous use of the Thealoz^®^ Duo eye drops exhibited a progressive attenuation of inflammation, with a very significant effect observed at T3 (Figure 4), with corresponding amelioration in the expression of a cluster of inflammatory proteins, namely protein S100A9, APOA1, Annexin A1 (ANXA1), SERPINA1, and Serpin B5 (SERPINB1) in all subgroups (Figure 5 and Supplementary Data S5). The expression of the DRYaqlip-specific inflammatory protein cluster, composed mainly of protein S100A8, High mobility group protein B1 (HMGB1), Mucin-5AC (MUC5AC), Vitronectin (VTN), and Protein deglycase DJ-1 (PARK7), was also significantly ameliorated at this prolonged time-point (Figure 5). Contrary to activated inflammatory processes, the interplay between another cluster of DAPs was predicted to be involved in the inhibition of apoptosis at T1 in all subgroups (DRY: z-score = −1.1; DRYlip: z-score = −1.6; DRYaqlip: z-score = −1.6) (Figure 4).

Notably, the high expression of proteins (e.g., Calpain small subunit 1 (CAPNS1), Argininosuccinate synthase (ASS1), Calpastatin (CAST), and Retinal dehydrogenase 1 (ALDH1A1)) involved in the inhibition of apoptosis at T1 in all the DED groups were rendered completely non-significant or significantly ameliorated at T3 following Thealoz^®^ Duo usage (Figure 4 and Figure 5). Likewise, the acute phase response signaling and organization of actin cytoskeleton, which were significantly affected at the baseline in both DRY and DRYlip, were virtually non-significant at the T3 time-point after the use of Thealoz^®^ Duo eye drops. On the other hand, in the DRYaqlip group with a more severe clinical picture, although these signaling mechanisms were not completely non-significant at T3 after Thealoz^®^ Duo treatment, a trend of gradual amelioration was observed, as evidenced by the decrement of the degree of significance (Figure 4). In general, the degree of significance of apoptosis, acute phase response signaling, synthesis of reactive oxygen species, complement activation, glycolysis, and organization of actin cytoskeleton was comparatively higher at T1 in DRYaqlip compared to both DRY and DRYlip subgroups. Strikingly, these biological functions were found to be gradually ameliorated at T3 (Figure 4). It is worth highlighting that the proteins involved in the regulation of antibacterial function consisted mainly of lacrimal-specific proteins such as LCN1, Lactotransferrin (LTF), Extracellular glycoprotein lacritin (LACRT), and LYZ (Figure 5), which were found to be not improved at both time-points following Thealoz^®^ Duo use.

## 4. Discussion

The present study combined both clinical assessment and the MS-based proteomics approach to decipher the hitherto unknown molecular changes in tears of DED patients that corroborate with the clinical parameters following the use of Thealoz^®^ Duo eye drops. In the first part of this study, the assessment of the clinical attributes of the DED patients demonstrated a significant improvement in the symptom anamneses based on the OSDI and VAS scores, as well as the TBUT, following the application of Thealoz^®^ Duo eye drops for ~56 days. Remarkably, the OSDI attribute showed the most significant improvement in DRY and DRYlip subgroups even after only 28 days of use of Thealoz^®^ Duo, with near-normal scores at the end of 56 days of continuous use of the eye drops. On the other hand, the improvement in the VAS score was only significantly recorded after 56 days of Thealoz^®^ Duo instillation in both DRY and DRYlip subgroups. These findings come as no surprise, as similar improvement patterns of the symptom anamneses, especially the OSDI parameter, were also demonstrated in DED patients after using Thealoz^®^ Duo eye drops, thereby corroborating the outcomes of previous clinical studies [12,13,15,31,32].

Moreover, the DRYlip subgroup showed a highly significant improvement in TBUT in addition to the OSDI and VAS scores at the 56-day time-point after the use of Thealoz^®^ Duo. The TBUT parameter is a key component for the clinical diagnosis of DED to assess the integrity of the lipid layer and stability of the tear film [33,34]. A study by Fariselli et al. has also documented similar improvement in the TBUT along with increased goblet cell density, as well as OSDI and VAS scores in DRYlip patients after the use of Thealoz^®^ Duo for 2 months [15]. On the contrary, no significant improvement in both OSDI and VAS attributes was demonstrated in the DRYaqlip subgroup at both time-points in our current study. Although there was a slight progressive improvement, the BST parameter, which represents the status of the lacrimal gland functionality, was found to be otherwise not significant after the application of Thealoz^®^ Duo. This finding is further substantiated by Chiambaretta et al., who also demonstrated that the BST was not significantly influenced even after 84 days of treatment with Thealoz^®^ Duo [32]. Nevertheless, the collective clinical results emerging from this study support the overt effectiveness of Thealoz^®^ Duo eye drops in ameliorating DED-related symptoms in the different subgroups.

The hallmark of this study is that the heightened inflammatory response in the DRYlip and DRY observed at T1 was ameliorated and completely abolished at T3 after the treatment with the eye drops. However, in the DRYaqlip group, although inflammation was not completely ameliorated following the use of Thealoz^®^ Duo, a relatively progressive reduction in the inflammatory response was observed at T3 in comparison to the untreated baseline. Inflammation is the main pathophysiological feature of DED, and it is characterized by the differential abundance of a specific cluster of protein markers [1,35,36]. Correspondingly, most of the well-known inflammatory proteins, namely S100A8, SERPINA1, ANXA1, and APOA1, were found to be significantly reduced in abundance in T3 compared to T1 in all the DED subgroups in this study [17,37,38,39,40,41]. Additional inflammatory response proteins, namely D-dopachrome decarboxylase (DDT), HMGB1, Lumican (LUM), PARK7, Plasminogen (PLG), and VTN, were found to be exclusively reduced in abundance in the DRYaqlip subgroup.

Similar to the inflammatory response, the expression of acute phase signaling proteins was found to be non-significant at T3 after the treatment of Thealoz^®^ Duo. It is recognized that the acute phase response is a rapid inflammatory response that provides protection using non-specific defense mechanisms [42]. Moreover, the correlation between the progression of many inflammatory diseases and tissue injury with oxidative stress-mediated signaling mechanisms and complement activation is well-recognized [43,44]. Hence, it is not surprising to observe heightened Reactive Oxygen Species (ROS) signaling in line with inflammatory response at T1 in the DRYlip and DRYaqlip subgroups in our present study, but was found to be decreased at T3 after the treatment with Thealoz^®^ Duo. Mounting studies have proposed that the organization of the actin cytoskeleton plays a central role in the epithelial barrier in regulating junctional integrity and remodeling under inflammatory pathological states [45,46]. This study also demonstrated the association between the inflammatory state on the ocular surface and significant activation in the organization of the actin cytoskeleton pathway in DED, and the efficacy of Thealoz^®^ Duo treatment in suppressing both pathways.

The current study has also shown that treatment with Thealoz^®^ Duo significantly hindered apoptotic cell death at T3 after the treatment with Thealoz^®^ Duo in all DED subgroups. Apoptosis is a normal multi-step process in physiological development to maintain cellular homeostasis [47,48]. Most of the anti-apoptotic proteins identified at T1 in DRY and DRYaqlip in this study have been described in previous studies, namely ALDH1A1, Peroxiredoxin-5 mitochondrial (PRDX5), Aldehyde dehydrogenase, dimeric NADP-preferring (ALDH3A1), Calpastatin (CAST), Argininosuccinate synthase (ASS1), Translationally-controlled tumor protein (TPT1), and Calpain small subunit 1 (CAPNS1) [17,37,41,49,50,51,52,53,54]. Another major highlight of this study is that the glycolysis pathway represented by Fructose-1,6-bisphosphatase 1 (FBP1), Glyceraldehyde-3-phosphate dehydrogenase (GAPDH), Malate dehydrogenase, cytoplasmic (MDH1), Phosphoglycerate mutase 1 (PGAM1), and Phosphoglycerate kinase 1 (PGK1) was significantly heightened at T2 compared to T1 in both DRY and DRYaqlip subgroups. Strikingly, all the aforementioned metabolic proteins were found to be non-significant at T3. Glycolysis, similar to apoptosis, is a highly conserved and multi-step process with a pivotal role in maintaining cellular homeostasis [48,55,56]. A recent study has provided a new insight into the molecular mechanism underlying the anti-inflammatory effect of FBP1, a high-energy intermediate of glycolysis, in effectively attenuating experimental arthritis [57]. Based on this observation, it is tempting to hypothesize that the instillation of Thealoz^®^ Duo triggered the activation of the glycolysis pathway to inhibit inflammation on the ocular surface, thereby restoring the homeostasis that was perturbed by the pathology.

In our study, the decreased abundance of TIMP1 in the tears of all the DRY, DRYlip, and DRYaqlip subgroups compared to CTRL was significantly improved with prolonged treatment with Thealoz^®^ Duo. The specific inhibitory property of TIMP1 on Matrix metalloproteinase-9 (MMP9) is well documented [58,59,60]. To date, MMP9 has been proposed to play a crucial role in the initiation and progression of DED by promoting corneal extracellular matrix degradation and epithelial cell loss [61,62,63,64]. Remarkably, in this study, the application of Thealoz^®^ Duo showed the therapeutic characteristic of the anti-inflammatory mechanism by promoting the expression of TIMP1 in all the DED subgroups.

It is noteworthy that the analysis without subdividing the DRY group into specific DRYlip and DRYaqlip subgroups hampers the evaluation of lacrimal gland function. Therefore, we divided the DRY into these two subgroups in this study, and further analysis using tears from the severe aqueous-deficiency group (DRYaqlip) enabled the precise detection and analysis of expression profiles of the known lacrimal-specific proteins such as PRR4, Proline-rich protein 1 (PROL1), LACRT, LYZ, and PIP [17,37,41,49,50,51,52,53,54]. The lacrimal-specific proteins possess antimicrobial and immunological properties on the ocular surface, and their abundance reflects the lacrimal gland functionality [17,65]. However, there was no increment observed in the abundance of these lacrimal proteins even after 56 days of Thealoz^®^ Duo treatment, which led to the conclusion that the eye drops do not have any direct effect on the functionality of this gland. Nevertheless, in this clinical study, the BST parameter was observed to be slightly improved at 56 days, and hence, the significant correlation between long-term use of Thealoz^®^ Duo (>3 months) and potential improvement in the lacrimal gland functionality requires further investigation.

Taken together, the formulation of Thealoz^®^ Duo clearly demonstrated a substantial improvement in the DED symptoms and quality of life features in all the DED subgroups. It is noteworthy that the functionally of the lipid layer in the tear film of evaporative DED patients attained healthy status with the treatment of Thealoz^®^ Duo. For the first time, proteomics investigation was instrumental in unraveling the molecular mechanistic pathways, including several novel aspects, associated with the efficacy of Thealoz^®^ Duo treatment for the duration of 56 days in all the DED subgroups. The acute instillation of Thealoz^®^ Duo eye drops orchestrated an activation of the metabolic pathway after ~28 days and ameliorated the vicious cycle of inflammation, acute phase response, synthesis of ROS, complement activation, and organization of actin cytoskeleton after a chronic treatment paradigm of ~56 days to promote the restoration of the ocular surface homeostasis. This study has also demonstrated the importance of combining clinical investigation with the MS-based proteomics platform to elucidate the distinct molecular changes underlying DED and its treatment, which are otherwise not manifested and measurable using only clinical diagnostic tools, particularly in the elusive aqueous-deficient DED.

## 5. Conclusions

In conclusion, this study has provided a comprehensive insight into the progressive mechanistic alterations in the tear proteome of different subgroups of DED patients associated with the therapeutic efficiency of Thealoz^®^ Duo eye drops at two different time-points, which supports the improvement in corresponding clinical attributes. The results of this investigation are envisioned to be a benchmark for effective clinical treatment of different DED subgroups in a time-dependent manner.

## Figures and Tables

**Table 1:** Descriptive statistics of the clinical attributes of the designated DED and healthy control groups (CTRL) at different time-points (T1–T3) following treatment with Thealoz^®^ Duo eye drops.

Groups	Samples (*n*)	Gender		Age		BST (mm)		TBUT (s)		OSDI		VAS	
		Female	Male	Mean	SEM	Mean	SEM	Mean	SEM	Mean	SEM	Mean	SEM
DRY vs. CTRL													
CTRL_T1	23	13	10	46.52	3.06	19.30	2.25	19.61	1.79	12.32	2.46	6.00	1.92
CTRL_T2	23	13	10	46.52	3.06	19.78	2.49	18.57	1.86	13.34	3.38	6.80	3.06
CTRL_T3	23	13	10	46.61	3.04	18.74	2.27	17.48	1.75	11.02	2.31	2.68	1.09
DRY_T1	35	22	13	57.60	2.48	11.29	1.65	5.09	0.95	46.53	3.01	34.40	3.55
DRY_T2	35	22	13	57.66	2.48	12.31	1.47	7.91	1.15	33.93	3.22	24.97	3.68
DRY_T3	34	21	13	57.88	2.54	13.53	1.50	10.44	1.32	25.37	2.80	17.16	2.94
DRYlip vs. CTRL													
CTRL_T1	15	9	6	42.67	4.17	24.53	2.46	20.47	2.35	11.82	2.97	3.72	1.48
CTRL_T2	15	9	6	42.67	4.17	25.00	2.92	19.87	2.54	9.92	2.68	2.29	1.06
CTRL_T3	15	9	6	42.80	4.15	23.87	2.58	19.73	1.91	10.96	2.87	1.86	0.93
DRYlip_T1	15	8	7	59.27	4.25	19.93	2.34	4.80	0.57	50.68	2.06	35.80	3.92
DRYlip_T2	15	8	7	59.27	4.25	19.67	1.69	9.20	1.77	36.08	4.20	28.27	5.95
DRYlip_T3	15	8	7	59.27	4.25	20.53	1.91	14.13	2.03	22.94	2.37	16.83	3.51
DRYaqlip vs. CTRL													
CTRL_T1	12	7	5	43.58	4.72	23.92	2.56	21.75	2.76	12.69	3.56	4.23	1.80
CTRL_T2	12	7	5	43.58	4.72	25.00	3.31	22.00	2.77	10.19	3.18	2.86	1.27
CTRL_T3	12	7	5	43.75	4.68	24.00	2.98	19.33	2.38	11.33	3.46	2.32	1.12
DRYaqlip_T1	12	8	4	57.92	2.93	3.42	0.42	6.25	2.67	38.98	7.67	31.07	7.75
DRYaqlip_T2	12	8	4	58.00	2.93	6.42	1.82	8.92	2.32	30.73	7.18	23.30	7.45
DRYaqlip_T3	12	8	4	58.00	2.93	7.75	1.68	9.00	2.17	25.37	6.92	16.95	6.77

Abbreviations: Basic Secretory Test (BST), Tear Breakup Time (TBUT), Ocular Surface Disease Index (OSDI) Questionnaire, Visual Analog Scale (VAS); Standard Error of the Mean (SEM), Moderate to Severe DED (DRY); subgroups: evaporative DED (DRYlip) and severe aqueous-deficient with evaporative DED (DRYaqlip), control groups (CTRL), different time-points (T1, baseline), (day 28 ± 4; T2) and (day 56 ± 4; T3).

## Data Availability

The original contributions presented in this study are included in the article/Supplementary Materials. Further inquiries can be directed to the corresponding author.

## Supplementary Materials

The following supporting information can be downloaded at: https://www.mdpi.com/article/10.3390/jcm14155525/s1, Data S1a: Clinical attributes of all the patients in the DRY vs. CTRL; Data S1b: Clinical attributes of all the patients in the DRYlip vs. CTRL; Data S1c: Clinical attributes of all the patients in the DRYaqlip vs. CTRL; Data S2a: Analysis of Variance (Wlech’s F-test; marked effects are significant at *p* < 0.05); Data S2b: Summary of the statistical analysis of all the clinical attributes [post-hoc Tukey honest significant difference (HSD) test for unequal N (Spjotvoll/Stoline test), marked effects are significant at *p* < 0.05]; Data S3a: MaxQuant Parameters; Data S3b: List of proteins identified in the DRY and CTRL groups at different time-points employing discovery proteomics strategy; Data S3c: List of proteins identified in the DRYlip and CTRL groups at different time-points employing discovery proteomics strategy; Data S3d: List of proteins identified in the DRYaqlip and CTRL groups at different time-points employing discovery proteomics strategy; Data S4a: List of the significantly differentially abundant proteins in the DRY vs. CTRL comparison analysis (Student’s *T*-test, *p*-value for highlighting < 0.05); Data S4b: List of the significantly differentially abundant proteins in the DRYlip vs. CTRL comparison analysis (Student’s *T*-test, *p*-value for highlighting < 0.05); Data S4c: List of the significantly differentially abundant proteins in the DRYaqlip vs. CTRL comparison analysis (Student’s *T*-test, *p*-value for highlighting < 0.05); Data S5a: List of the biological functions annotated to the significantly differentially abundant proteins in the designated DED subgroups comparison analysis [−Log (B-H *p* value < 0.05)]; Data S5b: List of the significantly differentially abundant proteins annotated to acute phase response in the designated DED subgroups comparison analysis; Data S5c: List of the significantly differentially abundant proteins annotated to apoptosis in the designated DED subgroups comparison analysis; Data S5d: List of the significantly differentially abundant proteins annotated to complement activation in the designated DED subgroups comparison analysis; Data S5e: List of the significantly differentially abundant proteins annotated to glycolysis in the designated DED subgroups comparison analysis; Data S5f: List of the significantly differentially abundant proteins annotated to inflammatory response in the designated DED subgroups comparison analysis; Data S5g: List of the significantly differentially abundant proteins annotated to antibacterial response in the designated DED subgroups comparison analysis; Data S5h: List of the significantly differentially abundant proteins annotated to organization of actin cytoskeleton in the designated DED subgroups comparison analysis; Data S5i: List of the significantly differentially abundant proteins annotated to organization of synthesis of reactive oxygen species in the designated DED subgroups comparison analysis.
